# Evaluating the Biocompatibility and Efficacy of Absorbable Three-Dimensional Micro-Nanofiber Scaffolds for Volume Restoration Following Post-Mastectomy Breast Reconstruction: An Experimental Study

**DOI:** 10.3390/jcm14113754

**Published:** 2025-05-27

**Authors:** Ji-Yeon Bae, JungHee Shim, Sunyoung Hwang, TaeHo Kim, BumMo Koo, Young Jin Lee, Ki Yong Hong, Chan Yeong Heo

**Affiliations:** 1 Department of Plastic and Reconstructive Surgery, Seoul National University Hospital, Seoul 03080, Republic of Korea; jita94@hanmail.net (J.-Y.B.); imyme5723@hanmail.net (S.H.); 2 Department of Plastic and Reconstructive Surgery, Seoul National University Bundang Hospital, Seongnam-si 13620, Gyeonggi-do, Republic of Koreajoanne2802@naver.com (B.K.); 3 Department of Research Administration Team, Seoul National University Bundang Hospital, Seongnam-si 13620, Gyeonggi-do, Republic of Korea; 4 OSFIRM R&D Center, H&BIO Co., Ltd., Seongnam-si 13605, Gyeonggi-do, Republic of Korea; taeho_91@naver.com (T.K.); yjlee@osfirm.co.kr (Y.J.L.); 5 Department of Plastic and Reconstructive Surgery, Seoul National University College of Medicine, Seoul 03080, Republic of Korea

**Keywords:** breast cancer, three-dimensional micro-nanofiber scaffold, breast reconstruction, breast-conserving surgery, clinical potential, preclinical experiment, rat

## Abstract

**Background/Objectives:** As the incidence of breast cancer increases, reliable, effective, and innovative solutions are required for breast deformities following breast-conserving surgery. We aimed to evaluate the biocompatibility and efficacy of optimized three-dimensional (3D) micro-nanofiber scaffolds and demonstrate their clinical potential through preclinical experiments. **Methods:** Seven-week-old male Sprague-Dawley rats were randomized into four groups. Group I (control group) received a 2-dimensional (2D) micro-nanofiber scaffold weighing 0.2 g; Groups II–IV received 3D micro-nanofiber scaffolds weighing 0.2, 0.3, and 0.6 g, respectively. These were subcutaneously implanted into the dorsal region and harvested with the surrounding tissues at 4, 8, and 16 weeks for histological evaluation. **Results:** The number of inflammatory cells was higher in Group IV than in Groups II and III at 4 weeks, with a significant increase in Group IV (*p* < 0.01) compared with that in Group I. At 8 weeks, it was significantly increased in Group III compared with that in Group I. Furthermore, at 16 weeks, it was significantly reduced in Group IV (*p* < 0.05) compared with that in Group I. The fibrosis depth in the 3D scaffolds revealed significant differences in Groups II, III, and IV (*p* < 0.001) compared with Group I at 4 weeks. The collagen fiber densities in the 3D groups were higher than those in the 2D group at 8 and 16 weeks. There were no statistically significant differences between the 3D groups. **Conclusions:** Absorbable 3D micro-nanofiber scaffolds enhance tissue integration and extracellular matrix formation following post-mastectomy breast reconstruction.

## 1. Introduction

Breast cancer is the most prevalent malignancy worldwide, affecting millions of women annually [1]. Despite advancements in treatment modalities that have contributed to improved survival rates, a significant postoperative concern persists regarding the potential for breast deformity, which can profoundly affect patients both physically and psychologically. Consequently, in recent decades, there has been a discernible shift toward favoring breast-conserving surgery (BCS) over mastectomy with the aim of preserving as much healthy breast skin as possible [2,3]. However, it is important to note that BCS may result in varying degrees of asymmetry, volume loss, and alterations in breast shape.

Traditionally, fat grafting has been the primary choice for reconstructive methods aimed at revising postoperative breast deformities by restoring volume and improving symmetry [4]. However, the effectiveness and durability of fat grafting can vary because of the resorption rates of grafted fat, which are influenced by multiple factors, including patient age, donor site of the fat tissues, graft technique, and irradiation of the recipient site, leading to unpredictable long-term outcomes [5]. Additionally, the availability of sufficient donor fat tissues may be limited in lean patients.

As the incidence of breast cancer increases, there is a continuous unmet need for innovative solutions that can reliably and effectively address breast deformities following BCS while minimizing donor-site morbidity through autologous tissue reconstruction and achieving long-lasting results. An innovative approach involves the development of absorbable scaffolds tailored specifically for breast tissue regeneration. Absorbable scaffolds represent a unique strategy for breast reconstruction by providing a temporary structural framework that supports tissue ingrowth and remodeling. These scaffolds can be engineered to mimic the natural extracellular matrix (ECM) of the breast tissue, thereby promoting cellular adhesion, proliferation, and differentiation. Additionally, they offer the advantage of gradual degradation over time, leaving behind regenerated tissues that seamlessly integrate with the surrounding tissues.

From the perspective of tissue engineering and regenerative medicine, absorbable scaffolds have the potential to revolutionize the field of breast reconstruction following malignancy treatment. In this study, we aimed to evaluate the biocompatibility and efficacy of optimized three-dimensional (3D) micro-nanofiber scaffolds (H&BIO Co., Ltd., Seongnam, Republic of Korea). The 3D micro-nanofiber scaffolds used in this study were fabricated via electrospinning using polycaprolactone (PCL), a well-established biodegradable and biocompatible polymer that has been extensively utilized in tissue engineering due to its favorable mechanical properties, tunable degradation kinetics, and Food and Drug Administration-approved safety profile [6,7,8].

Furthermore, we aimed to demonstrate the clinical potential of these scaffolds through preclinical experiments designed to mimic relevant physiological environments and assess their key parameters such as biocompatibility, inflammatory response, and tissue integration.

## 2. Materials and Methods

### 2.1. Scaffold Design and Fabrication

**Figure 1:**
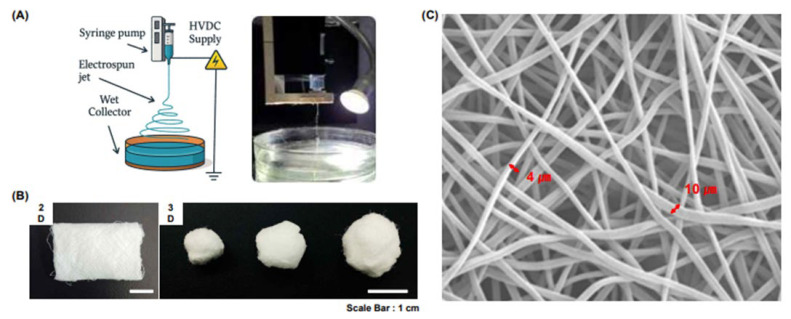
Schematic illustration and morphological characterization of 2D and 3D PCL micro-nanofiber scaffolds. (**A**) A schematic representation of the electrospinning process used to fabricate the PCL micro-nanofiber scaffolds. Scaffolds are provided in both 2D and 3D formats by H&BIO Co., Ltd., utilizing electrospinning techniques. (**B**) Representative morphological images of the fabricated 2D and 3D scaffolds. (**C**) SEM image showing the micro-nanofiber structure of the scaffold with fiber diameters ranging from 4 to 10 µm. Scale bar = 1 cm. 2D, two dimensional; 3D, three dimensional; SEM, scanning electron microscopy.

The 2D and 3D scaffolds (H&BIO Co., Ltd.) used in this study were fabricated via electrospinning using PCL (Figure 1a). A 15% (*w*/*v*) PCL solution was prepared by dissolving PCL in chloroform and used as the electrospinning precursor. The electrospinning process was performed under the following conditions: an applied voltage of 13 kV, a solution flow rate of 1 mL/h, and a distance of 10 cm between the nozzle and collector plate. To fabricate the 2D scaffold (Figure 1b), electrospinning was performed onto a dry collector plate, resulting in a flat, sheet-like structure. In contrast, the 3D scaffold was fabricated (Figure 1b) by electrospinning onto a moistened collector plate to promote the formation of a more voluminous, cotton candy-like architecture. To emulate the fibrous architecture of the native ECM, the electrospinning parameters were optimized to produce fibers with diameters ranging from 4 to 10 µm. Scanning electron microscopy (SEM) analysis confirmed the successful fabrication of fibers within the targeted diameter range (Figure 1c).

### 2.2. Experimental Design

Seven-week-old male Sprague-Dawley rats (Charles River Laboratories, Seoul, Republic of Korea) were randomly divided into four groups. Group I, the control group, received a 2D micro-nanofiber scaffold weighing 0.2 g, while Groups II–IV received 3D micro-nanofiber scaffolds weighing 0.2 g, 0.3 g, and 0.6 g, respectively (Table 1). Each scaffold was formed into a cylindrical shape with a diameter of 1 cm and height of 0.3 cm by packing the electrospun fiber mass into individual wells of a 12-well plate and securing the upper boundary using a silicone cap. Prior to use, all scaffolds were sterilized using ethylene oxide gas to ensure aseptic conditions for subsequent in vivo implantation. The scaffolds were subcutaneously implanted on the dorsal surface of the rats and harvested along with the surrounding tissues at 4, 8, and 16 weeks post-implantation.

### 2.3. Animal Model

Male Sprague-Dawley rats weighing 260–300 g were maintained on a 12-h light/dark cycle with ad libitum access to food and water. The rats were acclimated to their environment for a minimum of 1 week prior to use. All experimental procedures involving animals were conducted in accordance with the National Institutes of Health Guide for the Care and Use of Laboratory Animals and were approved by the Institutional Animal Care and Use Committee of Seoul National University Hospital (IACUC 22-0190-S1A0(2)). Subcutaneous dorsal incisions (1.5 cm) were made under general anesthesia (isoflurane), followed by blunt dissection, scaffold placement, and layered closure with 4-0 Vicryl and 4-0 nylon. Analgesics (buprenorphine) and antibiotics (enrofloxacin) were administered for 3 days post-surgery. All animals were euthanized at their respective time points (4, 8, 16 weeks) using CO_2_ asphyxiation in compliance with the institutional IACUC protocol.

Every effort was made to minimize animal suffering, reduce the number of animals used, and employ alternatives to in vivo techniques whenever available.

### 2.4. Histological Analyses

The scaffolds and surrounding tissues were extracted and treated with 4% paraformaldehyde for 24 h, followed by embedding in paraffin. Subsequently, tissues were sectioned into 5 µm thick slices and stained with hematoxylin and eosin (H&E) for general histological evaluation and Masson’s trichrome (MT) staining to assess collagen deposition. Hematoxylin- and eosin-stained slides were analyzed under a light microscope (Nikon, Tokyo, Japan) to evaluate the pathological changes, including cellular infiltration and inflammatory responses. Masson’s trichrome-stained slides were used to visualize collagen distribution, where collagen fibers appeared blue, the cytoplasm and muscle fibers appeared red, and the nuclei appeared black. Histological images were captured using a Leica SCN400 Image Viewer 12.3.3 (Leica Biosystems Imaging, Inc., Nussloch GmbH, Germany), and quantitative assessments of cell infiltration and collagen deposition were performed using computerized planimetry. Histological evaluation of the 3D micro-nanofiber scaffolds was conducted following the ISO standards (ISO 10993-6:2016) [9] with inflammatory cell types, including polymorphonuclear cells, neutrophils, lymphocytes, plasma cells, giant cells, and necrotic regions, systematically assessed.

Importantly, the 2D and 3D scaffolds were compared to assess the intended clinical application of the 3D scaffold in BCS. In BCS, volume maintenance and porosity are critical parameters that influence postoperative outcomes, such as tissue regeneration, volume retention, and cosmetic results. Therefore, comparing the newly developed 3D scaffold with a 2D counterpart, while keeping the material consistent, enabled us to isolate the effects of scaffold architecture and dimensionality in a manner directly relevant to the future clinical use of the scaffold in BCS.

To assess the impact of scaffold dimensionality on cell behavior, we evaluated the extent of cell infiltration into the PCL scaffolds.

### 2.5. Statistical Analyses

All values are presented as the means ± standard error of the mean. Given the small sample size and non-normal distribution of the data, Mann–Whitney U and Kruskal–Wallis nonparametric tests were employed to compare differences between independent groups. One-way analysis of variance was used for statistical analysis, and *p*-values < 0.05 were considered statistically significant. The data were categorized based on their *p*-values and are denoted as * *p* < 0.05, ** *p* < 0.01, and *** *p* < 0.001.

## 3. Results

### 3.1. Cell Infiltration into Scaffolds

**Figure 2:**
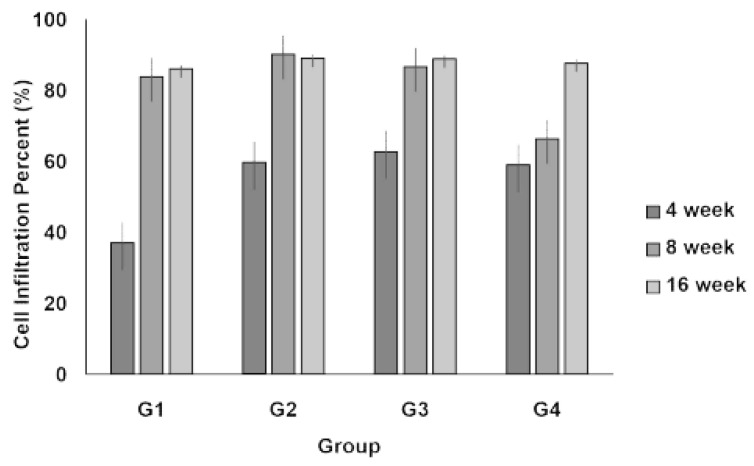
Infiltration of inflammatory cells. Cell infiltration is evaluated as the infiltration percentage (%) of the inflammatory cells and calculated using the following numerical formula: cell infiltration (%) = (implant area − clear area)/implant area × 100.

Cell infiltration into the implanted scaffolds was assessed over time (4, 8, and 16 weeks) for each group. The degree of cellular infiltration progressively increased in all groups over time, indicating that the host cells actively migrated into the scaffold structures (Figure 2).

At 4 weeks, Group I (2D scaffold) exhibited the lowest level of cell infiltration, whereas Groups II–IV (3D scaffolds) showed significantly higher levels. Infiltration was comparable among the 3D scaffold groups. After 8 weeks, a notable increase in cellular infiltration was observed across all groups. The 3D scaffold groups maintained a higher degree of infiltration than the 2D scaffold group, suggesting that the 3D structures facilitated better cell migration and integration with host tissues. At 16 weeks, cellular infiltration continued to progress and reached peak levels. The 3D scaffold groups showed a well-integrated cellular presence within the scaffold structures, with no substantial differences between Groups II, III, and IV. Meanwhile, although Group I (2D scaffold) exhibited increased infiltration compared to earlier time points, it remained lower than that of the 3D scaffold groups.

However, among the different 3D scaffold weights (0.2 g, 0.3 g, and 0.6 g), no significant differences were observed in infiltration levels, suggesting that increasing scaffold weight within this range does not substantially impact cellular infiltration.

### 3.2. Inflammation at the Interface of Scaffolds

**Figure 3:**
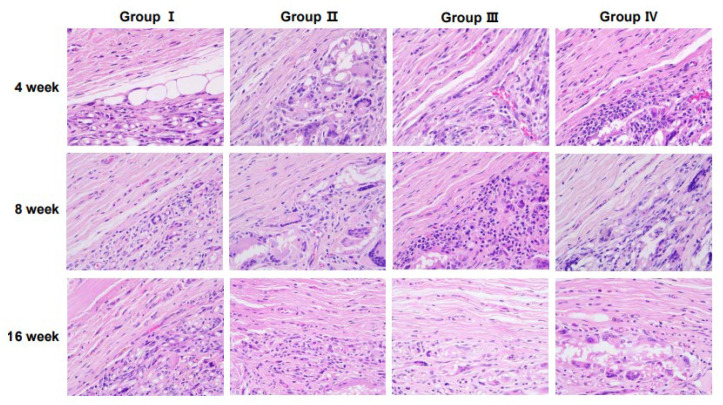
Histological analysis at the interface of scaffolds stained with hematoxylin and eosin. Groups I (2-dimensional [2D] micro-nanofiber scaffold) and II–IV (3-dimensional [3D] micro-nanofiber scaffolds) show inflammatory cells at 4, 8, and 16 weeks. (hematoxylin and eosin staining, 400× magnification).

Representative histological images of hematoxylin- and eosin-stained sections demonstrating tissue responses are shown in Figure 3. The number of inflammatory cells varied among groups over time, with notable differences observed at specific time points.

**Figure 4:**
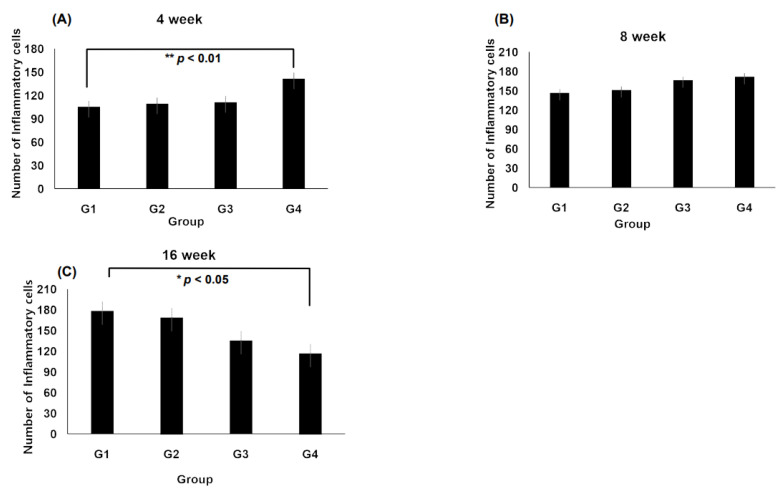
Number of inflammatory cells in each scaffold type along the time course. (**A**) The bar graph shows number of inflammatory cells at 4 week, which is higher in Group IV than in other groups. (**B**) The inflammatory cells are significantly increased in Group III at 8 weeks compared with that in Group I. (**C**) The number of inflammatory cells is significantly reduced in Group IV. The data are shown as the mean value ± standard error of the mean. *n* = 5 per group, * *p* < 0.05, ** *p* < 0.01 vs. group I.

At 4 weeks, Group IV exhibited a significantly higher number of inflammatory cells than Groups II and III, with a statistically significant increase observed compared with Group I (*p* < 0.01, Figure 4A). After 8 weeks, inflammatory cell infiltration remained prominent across all groups. Notably, Group III showed an increase in inflammatory cells compared with Group I (Figure 4B). At 16 weeks, a significant reduction in the inflammatory cell count was observed in Group IV compared with that in Group I (*p* < 0.05, Figure 4C), indicating a potential resolution of inflammation over time.

**Figure 5:**
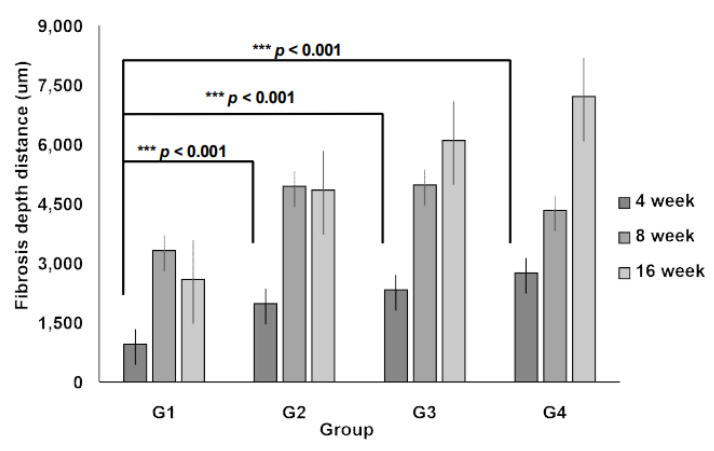
Fibrosis depth. The depths of the fibrosis patterns vary among the scaffold types. The data are shown as the mean value ± standard error of the mean. *n* = 5 per group, *** *p* < 0.001 vs. group I.

Additionally, the 3D micro-nanofiber scaffolds were histologically evaluated. At 4, 8, and 16 weeks, no significant differences were observed in the total number of inflammatory cell types (Table 2). Fibrosis depth within the 3D micro-nanofiber scaffolds showed significant differences among Groups II, III, and IV, with these groups displaying significantly greater fibrosis than Group I (*p* < 0.001, Figure 5), suggesting enhanced scaffold integration and ECM remodeling.

### 3.3. Collagen Regeneration Within Scaffolds

**Figure 6:**
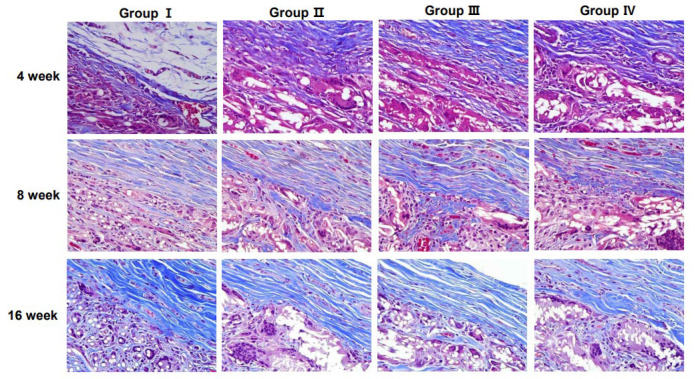
Histological analysis at the interface of scaffolds stained with Masson’s trichrome. Groups I (2-dimensional [2D] micro-nanofiber scaffold) and II–IV (3-dimensional [3D] micro-nanofiber scaffolds) show inflammatory cells at 4, 8, and 16 weeks. (Masson’s trichrome staining, 400× magnification).

Collagen content and distribution within the scaffolds were assessed using MT staining (Figure 6). The staining results demonstrated an increasing trend in collagen deposition over time, particularly in the 3D micro-nanofiber scaffold groups (Groups II, III, and IV).

**Figure 7:**
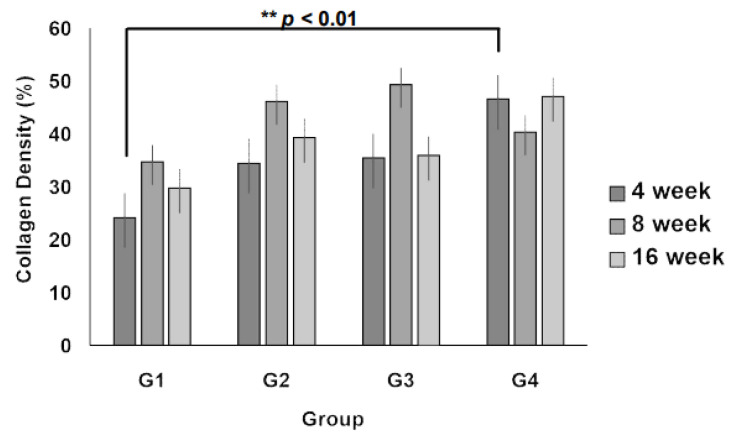
Collagen regeneration within the scaffolds. The collagen density displays collagen regeneration within the scaffolds, and the data are shown as the mean value ± standard error of the mean. *n* = 5 per group, ** *p* < 0.01 vs. Group I.

Quantitative analysis of collagen fiber density revealed that at 4 weeks, collagen deposition was significantly higher in Group IV than in Group I (*p* < 0.01, Figure 7), indicating enhanced ECM formation in the 3D scaffold with the highest weight (0.6 g). At 8 and 16 weeks, the collagen density in all 3D micro-nanofiber scaffold groups (Groups II, III, and IV) remained higher than that in the 2D scaffold group (Group I), although the differences were not statistically significant. Notably, there were no significant differences between the 3D scaffold groups, suggesting that increasing the scaffold weight beyond a certain threshold does not further enhance collagen deposition. These results indicate that 3D micro-nanofiber scaffolds promote collagen formation compared with 2D scaffolds, with the most pronounced effect observed at the early stage (4 weeks), particularly in the highest-weight scaffold (Group IV).

## 4. Discussion

BCS is a widely used surgical intervention for early stage breast cancer that allows patients to retain most of their breast tissue while achieving oncological safety [7]. However, BCS often results in volume loss, contour irregularities, and fibrosis, leading to aesthetic concerns and patient dissatisfaction. Traditional reconstructive approaches, including fat grafting and synthetic implants, have limitations, such as resorption, donor-site morbidity, and foreign body reactions [10,11].

The use of absorbable scaffolds is a promising alternative for promoting endogenous tissue regeneration. Recent studies have emphasized the potential of bioresorbable scaffolds to modulate immune responses and support soft tissue remodeling [12,13]. The biocompatibility of scaffold materials is crucial to ensure patient safety and minimize adverse effects such as chronic inflammation or fibrosis [14].

This study aimed to provide a safe and effective solution for breast deformities following BCS using absorbable scaffolds. To evaluate the biocompatibility and regenerative potential of absorbable 3D micro-nanofiber scaffolds, we assessed cell infiltration, inflammation, and collagen regeneration.

The observed cell infiltration patterns provided important insights into the biocompatibility and structural characteristics of the different scaffold types. Cell infiltration is a crucial factor in tissue regeneration and varies depending on scaffold structure and pore size. At 4 weeks, Group IV exhibited a tendency toward increased cell infiltration compared with Group I; however, this difference was not statistically significant (Figure 2). This suggests that while a higher scaffold volume may promote cellular ingress, the volume alone is insufficient to maximize infiltration. Thus, optimizing parameters, such as pore size, density, and physicochemical properties, is essential for enhancing cellular infiltration.

Inflammatory response is a key indicator of how implanted scaffolds interact with the host environment. At 4 weeks, Group IV showed a statistically significant increase in the presence of inflammatory cells compared with Group I, indicating that scaffold structural properties and volume differences may influence immune reactions (Figure 3). However, the reduction in inflammatory cells in Group IV at 16 weeks suggested tissue stabilization over time (Figure 4C). Additionally, at 8 weeks, Group III exhibited increased inflammatory cell infiltration compared with Group I, highlighting the role of the scaffold structure and material properties in modulating immune responses. These findings support the concept that scaffold architecture regulates macrophage polarization and downstream tissue outcomes [15] and the necessity of optimizing the scaffold design and degradation processes to minimize localized inflammation. Importantly, the absence of significant differences in inflammatory cell types across different time points (Table 2) implied that scaffold-induced inflammation was localized and did not trigger systemic immune responses.

Collagen fiber density is a critical indicator of ECM synthesis and scaffold-mediated tissue regeneration. At 4 weeks, Group IV exhibited a significantly higher collagen density than Group I, demonstrating its potential to promote ECM formation (Figure 6). Furthermore, the 3D micro-nanofiber scaffolds (Groups II, III, and IV) displayed higher collagen fiber density at 8 and 16 weeks than the 2D scaffold (Group I), although the differences were not statistically significant (Figure 7). These findings are in agreement with a previous study demonstrating the benefit of a 3D scaffold design in promoting soft tissue regeneration through sustained ECM remodeling [16].

Notably, Group IV, which had a relatively higher scaffold volume, exhibited a tendency toward increased ECM formation. However, the absence of significant differences among the 3D scaffold groups suggests that collagen deposition is influenced not only by structural changes but also by factors such as material stiffness, degradation rate, and surface chemistry [16]. Optimizing these parameters is critical for improving scaffold-based tissue regeneration. Additionally, the minimal variation in collagen fiber density among the 3D groups provides a useful reference for determining the maximum applicable volume of the scaffolds. This suggests that exceeding a certain volume threshold may not necessarily enhance ECM formation, emphasizing the importance of defining an optimal scaffold volume to maximize long-term tissue regeneration efficiency.

A recent study demonstrated that 3D-printed cell-delivery scaffolds can support regeneration across a variety of tissue types, underscoring their potential in applications such as breast reconstruction [17]. This approach represents a clinically translatable strategy for enhancing post-surgical soft tissue restoration by leveraging biomaterial-guided regeneration. Moreover, immunomodulatory biomaterials have emerged as a key component in this strategy, with the ability to actively modulate host immune responses and promote scaffold integration. By mitigating chronic inflammation and facilitating long-term tissue remodeling, these advanced biomaterials hold significant promise for improving clinical outcomes in reconstructive surgery [18]. We emphasize the need for comparison with standalone fat grafting [19,20], the lack of long-term human outcome data on volume retention, the limited indication scope for scaffold-based approaches (e.g., nipple-sparing mastectomies), and challenges related to cost-effectiveness and regulatory approval [21], which are limitations of the present study.

Further clinical studies are required to validate these findings and refine the scaffold design for optimized breast tissue regeneration following BCS.

## 5. Conclusions

This study found that the 3D micro-nanofiber scaffolds promoted cell infiltration, inflammation, and collagen regeneration in a scaffold- and time-dependent manner. Although these scaffolds showed greater cell infiltration and fibrosis depth than the 2D scaffolds at 4 weeks, these differences were not always statistically significant. Inflammation peaked at an early stage, but gradually decreased over time, indicating tissue stabilization. Collagen deposition was higher in the 3D scaffolds, with the largest increase observed in the scaffold with the highest volume. Despite minor variations among the 3D scaffolds, they consistently outperformed the 2D scaffold in promoting collagen regeneration, highlighting their potential for improved tissue integration and ECM formation.

## Figures and Tables

**Table 1:** Characterization of the groups.

Group	Type of Electrospinning	Weight (g)
Group I (G1)	2-dimensional (2D) micro-nano fiber scaffold	0.2
Group II (G2)	3-dimensional (3D) micro-nano fiber scaffold	0.2
Group III (G3)	3-dimensional (3D) micro-nano fiber scaffold	0.3
Group IV (G4)	3-dimensional (3D) micro-nano fiber scaffold	0.6

**Table 2:** Histological evaluation according to the ISO Standard.

	PMNs			Lymphocyte			Plasma Cell			Macrophage			Giant Cell			Necrosis		
	4 W	8 W	16 W	4 W	8 W	16 W	4 W	8 W	16 W	4 W	8 W	16 W	4 W	8 W	16 W	4 W	8 W	16 W
Group I	0	0	0	1	1	0.4	1	1	0.8	0	1	1	1	0.6	0.8	0	0	0
Group II	0	0	0	1	1	0.6	1	1	1	1	1	1	1	2	0.4	0	0	0
Group III	0	0	0	1	1	1	1	1	1	1	1	1	0.2	1.2	1.4	0	0	0
Group IV	0	0	0	1	1	1	1	1	1	0.6	0.8	1	0.8	1.4	1.4	0	0	0

## Data Availability

The data presented in this study are available on request from the corresponding author. The data are not publicly available due to consideration for the patients’ privacy.

## Abbreviations

The following abbreviations are used in this manuscript:

2D	Two-dimensional
3D	Three-dimensional
BCS	Breast-conserving surgery
ECM	Extracellular matrix
H&E	Hematoxylin and eosin
MT	Masson’s trichrome
PCL	Polycaprolactone
SEM	Scanning electron microscopy
